# Supplementary material for: The implementation of a standardized optimal procedure for peripheral venous catheters’ management: Results from a multi-dimensional assessment
Source: PLoS One. 2022 Jan 27;17(1):e0263227. doi: 10.1371/journal.pone.0263227 (PMC8794079; doi:10.1371/journal.pone.0263227)
Supplement: Attachment — Submitted filename: 2021-10-11 Response letter.docx [file pone.0263227.s003.docx]

**PlosOne**

**Submission ID: PONE-D-21-00993**

**Original Title: PERIPHERAL VENOUS ACCESS PROCESS and BEYOND: RESULTS FROM THE FIELD**

**Revised Title: THE IMPLEMENTATION OF A STANDARDIZED OPTIMAL PROCEDURE FOR PERIPHERAL VENOUS CATHETERS’ MANAGEMENT: RESULTS FROM A MULTI-DIMENSIONAL ASSESSMENT**

**Response to Editor and Reviewers**

Dear Editor and Reviewers,

We are very grateful for the response received concerning our manuscript, and the possibility to review and resubmit it, to the Journal.

We confirm our strong commitment to contribute to PlosOne.

Following your suggestions, we addressed the comments in a new version of the manuscript.

We hope to have addressed the revisions appropriately, responding comprehensively to your requests of integrations.

According to the Journal policy, we have submitted the followings:

- The present rebuttal letter.
- The revised manuscript, both in a clean and in a track-changes version.

We look forward to hearing from you.

With best regards,

The Authors

**Replies to the Editor**

Dear Editor,

Thank you for your kind comment and suggestion with regard to the modification of the title of our manuscript. According to your kind request, we have revised the title from “Organizational, efficiency and effectiveness impacts of a standardized optimal procedure, for the PVCs management” to “The implementation of a standardized optimal procedure for peripheral venous catheters’ management: results from a multi-dimensional assessment”.
