# Supplementary material for: The implementation of a standardized optimal procedure for peripheral venous catheters’ management: Results from a multi-dimensional assessment
Source: PLoS One. 2022 Jan 27;17(1):e0263227. doi: 10.1371/journal.pone.0263227 (PMC8794079; doi:10.1371/journal.pone.0263227)
Supplement: Attachment — Submitted filename: Response to Reviewer.docx [file pone.0263227.s004.docx]

**PlosOne**

**Submission ID: PONE-D-21-00993R2**

**Title: THE IMPLEMENTATION OF A STANDARDIZED OPTIMAL PROCEDURE FOR PERIPHERAL VENOUS CATHETERS’ MANAGEMENT: RESULTS FROM A MULTI-DIMENSIONAL ASSESSMENT**

**Response to Editor and Reviewers**

Dear Editor and Reviewers,

We are very grateful for the response received concerning our manuscript, and the possibility to review and resubmit it, to the Journal.

We confirm our strong commitment to contribute to PlosOne.

Following your suggestions, we addressed the comments in a new version of the manuscript.

We hope to have addressed the revisions appropriately, responding comprehensively to your requests of integrations.

According to the Journal policy, we have submitted the followings:

- the present rebuttal letter;
- the revised manuscript, both in a clean and in a track-changes version.

We look forward to hearing from you.

With best regards,

The Authors

**Replies to the Reviewer**

Dear Dr. Henry Egi Aloh,

We thank you for the revisions made to our manuscript.

According to your kind suggestions, we confirm we have revised the entire manuscript to make it presented in an intelligible fashion and written in standard English.

In addition, we have clarified and better explained the investigated technological scenarios, with specific detailed for Scenario 1 and Scenario 2.

Scenario 1 refers to the adoption of a “PVCs standardized optimal procedure”, that is the one suggested and implemented in the hospitals referring to Liguria Region (Italy) where the analysis was conducted. Scenario 1 refers to the simultaneous use of three specific medical devices suggested by the main guidelines available on the topic (O’Grady et al., 2011; RCN, 2010; INS, 2016; Scales, 2008): ChloraPrep (disposable device used for skin antisepsis), Nexiva (innovative PVC, to be inserted), and Posiflush (disposable device to be used for staying “in situ” and for washing up the patients after the catheter removal). The use of ChloraPrep, implemented for skin preparation, would rapidly kill micro-organism, thus also maintaining the antimicrobial activity for at least 48 hours (Florman et al., 2007; Crosby et al., 2001), and leading to a decrease in PCV associated infections’ occurrence rate (Manoury et al., 2008; McCann et al., 2016). The PVC Nexiva, in the comparison with traditional cannula, incorporates a stabilization platform, which could significantly reduce the risks of developing complications, such as phlebitis, infiltration/extravasation and dislodgement (Bausone-Gazda et al., 2010; Van Zundert, 2005). Beside an increased safety profile for patients, the use of the PVC Nexiva would simultaneously offer protection to the healthcare professionals, in terms of reducing the risk of exposure associated with needlestick injury (Lee et al., 2005). Using Posiflush at the same time, would reduce catheter-related bloodstream infections, thus improving standards of practice for catheter maintenance and management (Can & Yan, 2012).

The use of all the above mentioned three devices, should also be integrated with the following targets activities, to guarantee the implementation of the clinical procedure, as requested by the most diffuse Italian and European Guidelines (O’Grady et al., 2011; RCN, 2010; INS, 2016; Scales, 2008): i) number of insertion attempts lower than four; ii) PVC replacement in 96 hours; and iii) number of washes higher or equal to the number of PVC insertion.

On the contrary, Scenario 2 (i.e., not being in a standardized optimal procedure) refers to the use of only one or two out of the three disposable medical devices (thus using different devices for antisepsis, insertion, and washing-up phases), with target activities missing.

For being clearer and more comprehensive, we have produced the following table of synthesis.

|  | **Medical Devices used**   - ChloraPrep: Disposable device used for disinfection - Nexiva: innovative Peripheral Venous Catheter, to be inserted - Posiflush: disposable device for washing up the patients after the catheter removal | **Activities carried out**   - N. of insertion attempts <4 - Replace CVP in 96 hours - N. of washes ≥ N. of accesses to the catheter | **Being in procedure / Not being in procedure** |
| --- | --- | --- | --- |
| **SCENARIO 1** | Use of all the above devices | Performance of all the above target activities. | *Being in procedure* |
| **SCENARIO 2** | Use of only one or two, out of the above three devices (thus using other different consumables) | Target activities missing. | *Not being in procedure* |
